# Genome Sequences of Mycobacterium Strains Recovered from Captive Elephants with Tuberculosis

**DOI:** 10.1128/MRA.00671-21

**Published:** 2021-09-09

**Authors:** Evan P. Brenner, Syeda A. Hadi, Beth Harris, Suelee Robbe-Austerman, Srinand Sreevatsan

**Affiliations:** a Pathobiology & Diagnostic Investigation Department, College of Veterinary Medicine, Michigan State University, East Lansing, Michigan, USA; b National Veterinary Services Laboratories, Animal and Plant Health Inspection Services, USDA, Ames, Iowa, USA; Indiana University, Bloomington

## Abstract

Members of the Mycobacterium tuberculosis complex cause tuberculosis, infamous for enormous impacts on human health. As zoonoses, they also threaten endangered species like African/Asian elephants. We report the whole-genome sequences of Mycobacterium tuberculosis bv. tuberculosis and *Mycobacterium tuberculosis* bv. bovis from two zoo elephants in the United States.

## ANNOUNCEMENT

Two captive elephants from United States zoos presented with unrelated cases of tuberculosis. Postmortem isolation yielded Mycobacterium tuberculosis bv. bovis (MBO) in one case from Illinois in 1997 (isolate A2) and Mycobacterium tuberculosis bv. tuberculosis (MTB) in the other from the Washington, DC, area in 2000 (isolate A4). The isolates were confirmed using spoligotyping and a MassARRAY-based targeted single-nucleotide polymorphism (SNP) analysis (1). Both isolates were cultured and maintained by the USDA National Veterinary Services Laboratories (NVSL) using standard mycobacterial methods as described by Joshi et al. (1), and DNA was provided to our lab for genome analysis. The DNA was submitted for whole-genome sequencing (WGS) by Novogene (CA, USA).

Novogene prepared paired-end libraries (2 × 150 bp) using the NEBNext DNA library prep kit (New England BioLabs). The quality was assessed using a Qubit fluorometer, an Agilent Bioanalyzer instrument, and quantitative PCR (qPCR) before sequencing using the Illumina NovaSeq 6000 platform. Novogene performed trimming and quality control (QC) using in-house software (v1.0), discarding any read pairs that contained contaminant adapter sequences after trimming, as well as any pairs where a read contained ≥10% N nucleotide calls or a Phred score of ≤5 before data delivery to Michigan State University (MSU).

Unless noted, all software was used with default parameters. The genome coverage was calculated using HISAT2 (2) to align the reads against MTB H37Rv (GenBank accession number NC_000962.3) and MBO AF2122/97 (LT708304.1) as references for A4 and A2, respectively. The sequences were contamination screened using Kraken2 (3) with the standard database and were 98.9%+ classified as Mycobacterium tuberculosis complex. All reads regardless of classification were used for *de novo* genome assembly using Shovill v1.1.0 (4) (-gsize 4.35M [MBO] or 4.41M [MTB], -minlen 300). The contigs were corrected and scaffolded using RagTag v1.1.1 (5) with M. tuberculosis H37Rv and M. bovis AF2122/97 as the respective references. The scaffolded output was submitted to NCBI for annotation using the Prokaryotic Genome Annotation Pipeline (PGAP) (6).

Out of 15,968,006 MBO isolate A2 reads, 93% mapped to AF2122/97, yielding about 515× coverage. *De novo* assembly produced 16 contigs, with an *N*_50_ value of 4,301,900 bp, 65.61% GC content, and a total sequence length of 4,316,405 bp, and PGAP annotated 3,981 coding DNA sequences (CDSs), 3 rRNAs, 45 tRNAs, 3 noncoding RNAs (ncRNAs), and 188 pseudogenes.

Out of 14,440,792 MTB isolate A4 reads, 85% mapped to H37Rv, yielding about 494× coverage. *De novo* assembly produced 26 contigs, with an *N*_50_ value of 4,363,477 bp, 65.58% GC content, and a total sequence length of 4,380,121 bp, and PGAP annotated 4,074 CDSs, 3 rRNAs, 45 tRNAs, 3 ncRNAs, and 158 pseudogenes.

Tuberculosis in elephants is poorly studied, and these genome sequences provide visibility into lethal threats for these animals. Zoo elephants face unique and varied exposures to humans, animals, and each other in captivity, and genomic investigation of these newly sequenced genomes allows novel insight into transmission and pathogenesis in these endangered species in captive settings.

### Data availability.

The FASTQ files are available through NCBI under BioProject accession number PRJNA729093. The raw reads can be found under SRA accession numbers SRR14508917 (MBO strain A2) and SRR14508916 (MTB strain A4). The post-PGAP sequences can be found under accession numbers JAHCXD000000000 (MBO strain A2) and JAHCXC000000000 (MTB strain A4).
